# Compound KTI-2338 Inhibits ACVR1 Receptor Signaling in Fibrodysplasia Ossificans Progressiva

**DOI:** 10.3390/pharmaceutics17121590

**Published:** 2025-12-10

**Authors:** Neeltje M. Rosenberg, Lidiia Zhytnik, Lisanne E. Wisse, Esmée Botman, Jennifer L. Lachey, E. Marelise W. Eekhoff, Dimitra Micha

**Affiliations:** 1 Department of Internal Medicine, Section Endocrinology and Metabolism, Amsterdam UMC Location Vrije Universiteit Amsterdam, De Boelelaan 1117, 1081 HV Amsterdam, The Netherlands; 2 Rare Bone Disease Center Amsterdam, 1081 HV Amsterdam, The Netherlands; 3 Amsterdam Reproduction and Development Research Institute, 1105 AZ Amsterdam, The Netherlands; 4 Amsterdam Movement Sciences, Musculoskeletal Health, Tissue Function & Regeneration, 1081 HV Amsterdam, The Netherlands; 5 Department of Human Genetics, Amsterdam UMC Location Vrije Universiteit Amsterdam, De Boelelaan 1117, 1081 HV Amsterdam, The Netherlands; 6 Keros Therapeutics, Inc., 1050 Waltham Street, Lexington, MA 02421, USA

**Keywords:** fibrodysplasia ossificans progressiva, fibroblasts, activin A, BMP, heterotopic ossification, ACVR1, ALK2 inhibitor

## Abstract

**Background/Objectives:** Fibrodysplasia Ossificans Progressiva (FOP) is a rare genetic bone disorder, leading to progressive immobilization through the formation of bone in muscles, tendons, and ligaments. A variant in the *ACVR1* gene results in a constitutively overactive ALK2 receptor, leading to the aberrant activation of the SMAD1/5/9 pathway. This activation occurs not only in response to Activin A, which does not normally activate this pathway, but also through heightened sensitivity to BMP ligands and even in the absence of ligand binding. This dysregulated signaling ultimately drives the formation of heterotopic ossification. The inhibition of the altered ALK2 receptor holds promise as a potential treatment strategy that is currently being investigated in several trials. In this study, we performed an in vitro characterization of novel kinase inhibitor KTI-2338 with high selectivity for the ALK2 receptor. **Methods:** Dermal human FOP and control fibroblasts were cultured in osteogenic medium with and without the inhibitor to assess the effect on transdifferentiation into osteoblast-like cells. **Results:** Compound KTI-2338 elicited effects consistent with inhibiting aberrant Activin A signaling and receptor sensitization, through reductions in osteogenic markers and pSMAD1/5/9 expression levels. In line with this, a pattern of reduced Alizarin Red staining was observed following treatment with the compound, indicating reduced mineralization. **Conclusions:** These findings indicate that kinase inhibitor KTI-2338 disrupts the pathological processes underlying FOP and may offer a new therapeutic option for this devastating disease.

## 1. Introduction

Fibrodysplasia Ossificans Progressiva (FOP; OMIM #135100) is an ultra-rare bone disease that occurs in 1 per 1.5–2 million people [1,2]. It is characterized by heterotopic ossification (HO), the progressive formation of bone tissue outside the skeleton, in fibrous tissues such as muscle, ligaments, and tendons. At birth, the only sign of FOP is the deformity of the toes [3,4]. Flare-ups may begin appearing around the age of four years, characterized by inflammatory soft tissue swellings [5]. These may occur spontaneously or can be triggered by trauma or (virus) infections. Even though some flare-ups may resolve completely, most result in the formation of HO [6]. Patients become progressively immobilized by joint ankylosis and loss of muscle function; by the age of twenty years, they frequently face wheelchair dependance [5]. HO around the thorax can lead to thoracic insufficiency syndrome (TIS), a common complication that significantly shortens the life expectancy of FOP patients [7].

The Transforming Growth Factor beta (TGFβ) superfamily of ligands and receptors are crucial mediators of cell differentiation and tissue homeostasis, including bone tissue. It comprises >35 ligands which signal through heterotetramers of type I and type II receptor dimers. In general, Bone Morphogenetic Proteins (BMPs) bind type I receptors, such as activin-like kinase 2 (ALK2), which initiates an intracellular signaling cascade through the phosphorylation of SMAD1/5/9 [8]. In contrast, Activin A has affinity for activin type II receptors and signals through the phosphorylation of SMAD2/3 [9]. Notably, Activin A can bind the type I ALK2 receptor; however, rather than activating, it forms a non-signaling complex with the receptor that inhibits BMP signaling. Pathogenic variant NM_001105.4:c.617G>A (p.Arg206His) is found in 95% of FOP patients. It is a single nucleotide change, caused by a guanine to adenine substitution (c.617G>A), resulting in the replacement of arginine with histidine at codon 206 of the Activin A receptor type 1 (*ACVR1*; OMIM #102576) gene [10]. This causes an alteration in the glycine–serine-rich domain (GS domain) of the ACVR1/ALK2 receptor, resulting in aberrant osteogenic SMAD1/5/9 activation by the mutated receptor in response to Activin A binding [11]. In addition, the receptor is hyperresponsive to BMP activation, leading to increased pSMAD1/5/9 signaling [12]. A third mechanism of aberrant signaling by the mutated receptor is attributed to its ligand-independent activation [13,14]. Together, this exaggerated osteogenic signaling ultimately results in HO formation.

Considering the well-characterized significance of aberrant ACVR1/ALK2 signaling in FOP pathology, several trials are in progress targeting components of this pathway; these include Activin A (NCT05394116) or the ACVR1 receptor (NCT04307953, NCT05039515, NCT05090891). No treatments for FOP have yet been approved in Europe to prevent flare-ups or HO. Although palovarotene is approved as the first treatment to prevent HO in FOP in the USA, Canada, Australia, and Russia, questions regarding its safety and efficacy have resulted in its disapproval by the European Medicines Agency [15]. KTI-2338 is a novel ALK2 inhibitor developed for the treatment of diseases characterized by hyperresponsive ALK2 signaling [16,17]. It is a derivative of a pan BMP receptor inhibitor LDN-193189 and is highly selective for the ALK2 receptor (Figure S1) [18]. Parent molecule LDN-193189 demonstrated efficacy in preventing ectopic bone formation in the Q207D mouse model of FOP [19]. To evaluate the novel inhibitor KTI-2338 for its ability to attenuate ALK2 activity in a relevant human system, we employed an in vitro model using patient-derived fibroblasts, enabling the study of the HO process in FOP.

Since bone biopsies may cause HO, it is challenging to safely derive bone cells from FOP patients [20]. Micha et al. identified an alternative method involving the direct conversion of primary human fibroblasts into osteogenic cells through transdifferentiation [21]. This approach circumvents the intermediate step of converting fibroblasts into induced pluripotent stem cells (iPSCs). In this model, dermal fibroblasts from FOP patients with the *ACVR1* classical variant undergo enhanced transdifferentiation into osteogenic cells compared to healthy controls, using platelet lysate-based osteogenic media. It models increased pSMAD1/5/9 stimulation and transcriptional regulation in response to both BMPs and Activin A in human FOP cells [22]. This primary cell model was used to investigate the effect of compound KTI-2338 as a new treatment option in FOP.

## 2. Methods

### 2.1. Patient Characteristics

Fibroblasts were derived through skin biopsies, from five FOP patients and five healthy controls. Informed consent was obtained before study inclusion according to guidelines of the VU University Medical Centre. Patient characteristics have been described by De Ruiter et al. [22] and Micha et al. [21] and are summarized in Table 1. All FOP patients were confirmed to carry the *ACVR1* (c.617G>A; R206H) variant. Age at biopsy varied from 14 to 62 years, and three male and two female FOP patients were included. Three patients (P2, P4, P5) experienced a flare-up at the time of biopsy, for which they received treatment from at least one week before to one week after the biopsy. This included nonsteroidal anti-inflammatory drug (NSAID) treatments and medication for pain relief. At the biopsy site, no flare-ups were noted and no HO was formed after one year.

### 2.2. Cell Culture and Treatments

Cells were routinely cultured in Ham F10 media (Gibco, Grand Island, NY, USA, 31550-031) supplemented with 10% fetal calf serum (FCS; Gibco, Grand Island, NY, USA, 10270-106) and 1% penicillin/streptomycin (Gibco, Grand Island, NY, USA, 15140-122) in humidified conditions at 37° with 5% CO_2_. For cell viability assessment, 223 nM of KTI-2338 or the equivalent DMSO concentration was used to treat four FOP and four control cell lines on days 7, 14, and 21 with CellTiter-Blue (Promega, Madison, WI, USA, G8080). Three FOP cell lines were used to investigate the expression of the inhibitor of DNA binding 1 (*ID1*) and 2 (*ID2*). These cells were treated for six hours either with Activin A (62.5 ng/mL; Sigma Aldrich, St Louis and Burlington, MA, USA, A4941-10UG), Activin A and DMSO (Sigma-Aldrich, St Louis and Burlington, MA, USA, D2650), or Activin A and inhibitor KTI-2338 (Molecular weight: 447.52) at 2.23 nM, 22.3 nM, or 223 nM. In two FOP cell lines (P1, P4), Western blot was performed to assess pSMAD1/5/9 expression. These cells were subjected for one hour to Activin A treatment, in combination with a 2.23 nM, 22.3 nM, or 223 nM inhibitor, after overnight FCS starvation.

### 2.3. Western Blot

NuPAGE^®^ LDS Sample Buffer (Invitrogen, Carlsbad, CA, USA, NP007) was used to prepare whole cell lysates; protein was transferred to a nitrocellulose membrane (Invitrogen Carlsbad, CA, USA). The membrane was blocked by incubating in blocking buffer for one hour (LI-COR Biosciences, Lincoln, NE, USA, 92770001), followed by overnight primary antibody incubation against pSMAD1/5/9 (cell signaling; Cat#13820S, Danvers, MA, USA) and Actin (Abcam; Cat#ab14128, Cambridge, UK). Secondary antibodies were applied for one hour (IRDye 800 CW goat anti-rabbit IgG and IRDye 680 CW goat anti-mouse; LI-COR Biosciences, Lincoln, NE, USA). The Odyssey scanning system was used to visualize fluorescence (Odyssey version 4, LI-COR Biosciences, Lincoln, NE, USA). The intensities of the pSMAD1/5/9 bands were quantified using ImageJ software v.1.54 (Bethesda, MD, USA) and normalized based on corresponding Actin band intensities.

### 2.4. Osteogenic Transdifferentiation

To further investigate the osteogenic properties of cells treated with KTI-2338, we performed osteogenic transdifferentiation with five FOP and five control cell lines of the cultured fibroblasts. A total of 100,000 cells per well were seeded in 6-well plates for 28 days during which they received osteogenic medium supplemented with 223 nM inhibitor, or osteogenic medium alone or standard medium as described above. The osteogenic differentiation medium consisted of minimal essential medium alpha (α-MEM+GlutaMAX; Gibco, Grand Island, NY, USA, 32561-029), 90 µg/mL 2-phospho-L-ascorbic acid trisodium (Sigma, Burlington, MA, USA, 49752-10G), 5 mM β-glycerophosphate disodium salt hydrate (Sigma, Burlington, MA, USA, G5422-100G), 0.2% *v*/*v* Heparin (LEO Pharma, Amsterdam, The Netherlands), 5% *v*/*v* human platelet lysate (Stemcell technologies, Zwolle, The Netherlands, 06962), and 1% *v*/*v* penicillin/streptomycin (Gibco, Grand Island, NY, USA, 15140-122). Every 3–4 days, the medium was refreshed for 14, 21, and 28 days.

### 2.5. Reverse Transcription Quantitative Polymerase Chain Reaction (RT-qPCR)

The quantification of gene expression was performed using RT-qPCR. *ID1* and *ID2* expression levels were assessed in FOP fibroblasts after the treatment with Activin A with or without KTI-2338. In the transdifferentiated osteogenic cells, the gene expression of alkaline phosphatase (*ALP*), runt-related transcription factor 2 (*RUNX2*), *ID1*, and inhibin A (*INHBA*) were measured on days 14 and 21. RNA was isolated with the Zymo Quick-RNA kit (Zymo research, Irvine, CA, USA, R1055). The VILO cDNA synthesis kit (Invitrogen, Carlsbad, CA, USA, 11754050) was employed to reverse transcribe cDNA using total 140 ng of RNA. Then, RT-qPCR was performed in the total volume of 10 μL in duplicate, with 1 pmol/µL primers (Integrated DNA technologies, IDT, Coralville, IA, US); primers are listed in Table 2. The average in the duplicate wells was used per sample for the analysis. For the measurement of gene expression, standard methods were used with the LightCycler 480 (Roche Diagnostics, Basel, Switzerland). Gene expression was normalized by comparison with tyrosine 3-monooxygenase/tryptophan 5-monooxygenase activation protein zeta (*YWHAZ*) expression. The calculation of the relative gene expression was based on the ΔΔCt method.

### 2.6. Alkaline Phosphatase and Alizarin Red Staining

Staining for ALP activity was performed after 21 days of osteogenic transdifferentiation cell culture. AR staining took place after 21 and 28 days for the detection of calcium deposits. For the staining, a total of 30,000 cells were seeded in 12-well plates. Cells underwent fixation with 4% paraformaldehyde in phosphate-buffered saline (DPBS; Gibco, Grand Island, NY, USA, 14190-094) for fifteen minutes, followed by rinsing with deionized water for three times. After this, staining was performed with the NBT/BCIP solution bioReagent (Sigma-Aldrich, St. Louis, MO, USA, B6404-100 mL) 0.5 mL per well for 40 min or 2% AR solution (Sigma-Aldrich, St. Louis, MO, USA, A5533-25G) with 1 mL per well for at least 20 min. Excess solution was washed away with deionized water four times. The quantification of ALP and AR was conducted with ImageJ Fiji software v.1.54 (Bethesda, MD, USA) by calculating the amount of staining in a representative area.

### 2.7. Statistical Analysis

Expression of *ID1* and *ID2* was compared with one-way ANOVA and Dunnett’s multiple comparisons test. To assess cell viability, two-way ANOVA and Dunnett’s multiple comparisons test were employed to compare FOP and control cells at different timepoints. Two-way ANOVA, combined with a Sidak multiple comparisons test, was also used for the comparison of *ALP*, *RUNX2*, *ID1*, and *INHBA* expression on days 14 and day 21, as well as to compare ALP and AR staining levels after quantification. A *p* value of <0.05 was considered to be statistically significant. The stated statistical analyses were conducted with GraphPad Prism 10.2 (GraphPad Software Inc., San Diego, CA, USA).

## 3. Results

### 3.1. Inhibitor KTI-2338 Decreases Activin A-Stimulated pSMAD1/5/9 Signaling in FOP Fibroblasts

**Figure 1:**
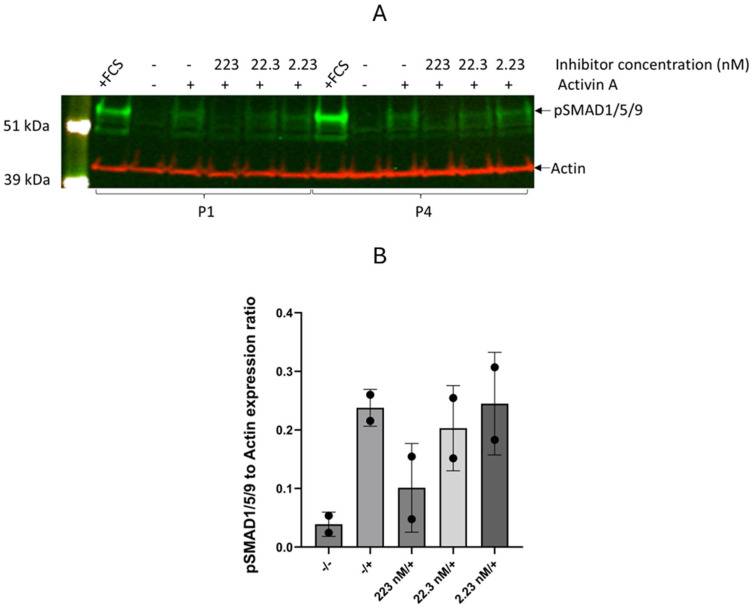
Western blot analysis of pSMAD1/5/9 expression in two FOP cell lines (P1, P4). (**A**) After overnight FCS starvation, fibroblasts from FOP patients P1 and P4 were treated with Activin A in combination with a concentration range of KTI-2338 for one hour. Samples with added FCS are indicated as +FCS. The expression of pSMAD1/5/9 was normalized based on Actin. (**B**) Quantification of pSMAD1/5/9 expression in the two FOP cell lines normalized to Actin. Symbols represent values for each cell line; error bars indicate mean ± standard deviation.

In order to investigate the effect of the inhibitor on aberrant SMAD activation by Activin A, the FOP fibroblasts of two patients (P1 and P4) were treated with Activin A and the inhibitor at concentrations of 2.23 nM, 22.3 nM, or 223 nM. Western blot analysis showed higher expression of pSMAD1/5/9 in cells treated with Activin A (Figure 1). This expression was decreased by the inhibitor at 223 nM. Lower concentrations of the inhibitor did not affect pSMAD1/5/9 expression.

### 3.2. Inhibitor KTI-2338 Decreases Activin A-Stimulated Expression of TGFβ Superfamily-Regulated Genes in FOP Fibroblasts

**Figure 2:**
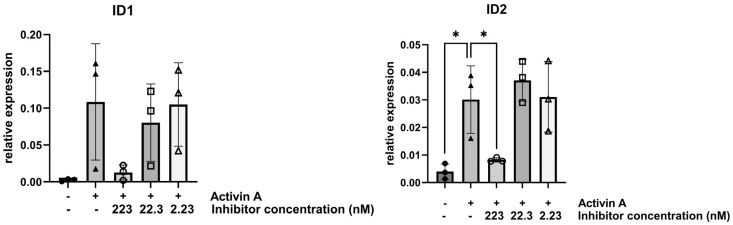
*ID1* and *ID2* expression in FOP fibroblasts treated for six hours with Activin A and different concentrations of KTI-2338. Cells from three FOP patients (P1, P4, P5) were used. Gene expression was normalized to *YWHAZ*. Symbols represent value for each cell line; error bars indicate mean ± standard deviation; significant differences are indicated by an asterisk (* *p* < 0.05).

In order to assess the effect of KTI-2338 on Activin A-mediated gene expression of SMAD1/5/9 target genes, FOP fibroblasts (P1, P4, P5) were treated for six hours with Activin A or with the addition of the inhibitor at concentrations of 2.23 nM, 22.3 nM, or 223 nM. Cells stimulated with Activin A showed up to a 40-fold higher expression of *ID1*; however, upregulation was variable between cell lines and did not reach statistical significance. Activin A treatment significantly upregulated *ID2* expression by up to 7-fold (*p* = 0.011; Figure 2).

Interestingly, the incubation of Activin A-treated FOP cells with KTI-2338 (223 nM) led to a marked reduction in *ID1* levels, although the decrease was not statistically significant. KTI-2338 (223 nM) significantly reduced *ID2* expression to levels that approached that of untreated fibroblasts (*p* = 0.031). Lower concentrations (2.23 nM and 22.3 nM) failed to show a reduction in *ID1* and *ID2* expression in agreement with no changes in pSMAD1/5/9 (Figure 1 and Figure 2).

### 3.3. Cell Viability Is Unaffected by the Inhibitor

**Figure 3:**
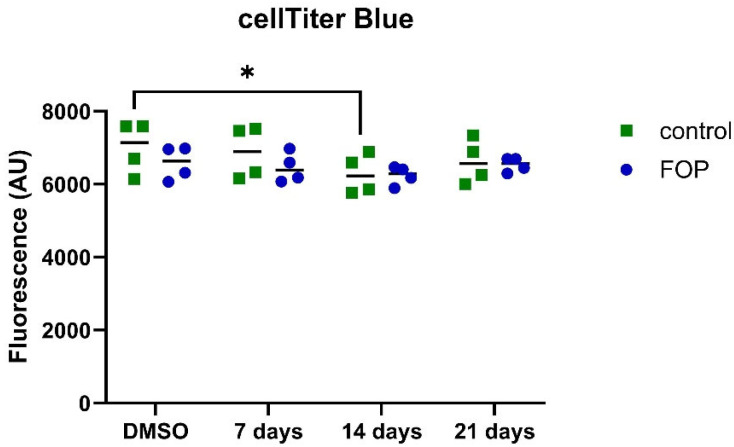
Cell viability in four FOP (P1, P2, P3, P5) and four control (C1, C2, C3, C5) cell lines was measured after 7, 14, and 21 days of treatment with the ACVR1 inhibitor at 223 nM compared to cells treated with the equivalent DMSO concentration. Symbols represent the value for each cell line, lines represent medians, and significant differences are indicated with an asterisk (* *p* < 0.05).

To rule out the possibility of inhibitor toxicity, a cell viability assay was performed 7, 14, and 21 days after treatment with 223 nM of the inhibitor. There was no strong reduction in the cell viability of the patients (P1, P2, P3, P5) or control cells (C1, C2, C3, C5) over the time course of 21 days (Figure 3). Control cells showed a transient reduction after 14 days of treatment with the inhibitor compared to untreated cells. However, FOP and control cells did not show a significant reduction at any other timepoint. For the transdifferentiation experiments, we therefore used the 223 nM concentration of the inhibitor.

### 3.4. Expression of Osteogenic Differentiation Genes RUNX2, ID1, and INHBA Decreased in FOP Cells After Inhibitor Treatment

**Figure 4:**
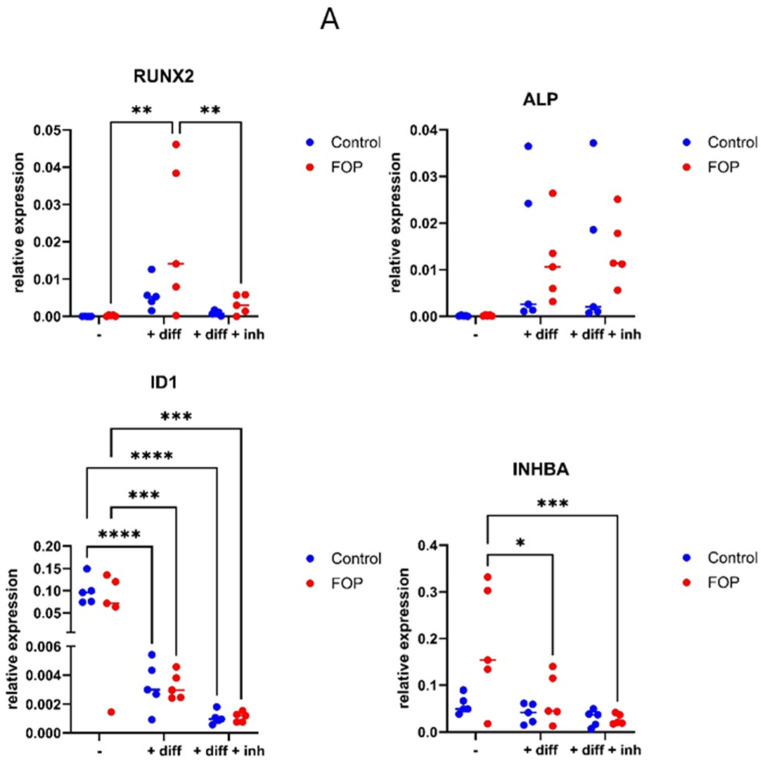

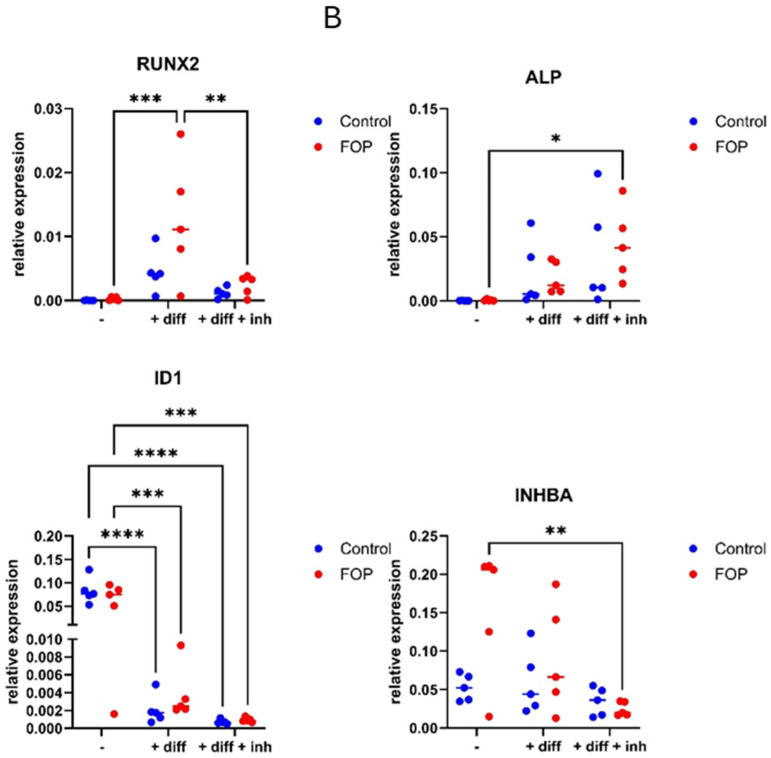
Effect of the inhibitor (223 nM) on the osteogenic transdifferentiation of five FOP (P1–P5) and five control cells (C1–C5). (**A**) After 14 days, the relative expression of *RUNX2*, *ID1*, and *INHBA* in FOP cells is reduced in the presence of the inhibitor. (**B**) Relative expression of *RUNX2*, *ALP*, *ID1*, and *INHBA* after transdifferentiation on day 21. *RUNX2*, *ID1*, and *INHBA* expression is reduced by inhibitor treatment. Cells were untreated (-), treated with osteogenic medium (+ diff), or treated with osteogenic medium and KTI-2338 (+ diff + inh). Circles represent values for each cell line, lines represent medians, and significant differences are indicated with an asterisk (* *p* < 0.05, ** *p* < 0.01, *** *p* < 0.001, **** *p* < 0.0001). Alkaline phosphatase and Alizarin Red staining are reduced by the inhibitor.

The effect of the inhibitor on the osteogenic transdifferentiation of FOP (P1-P5) cells was examined based on osteogenic gene expression on days 14 and 21. For this purpose, 223 nM KTI-2338 was used based on the ability of the inhibitor to inhibit pSMAD1/5/9 as well as ID1 and ID2 expression. *RUNX2* expression in FOP cells increased significantly both on days 14 and 21 after osteogenic transdifferentiation. After KTI-2338 treatment, *RUNX2* significantly decreased in FOP cells at both timepoints (Figure 4, Table S1). *ALP* expression increased after osteogenic transdifferentiation; however, no significant differences were observed between FOP and control cells or after inhibitor treatment compared to the differentiated cells. At both timepoints, *ID1* expression significantly decreased in both FOP and control cells following transdifferentiation compared to their undifferentiated counterparts. Considering the low ID expression level, treatment with KTI-2338 did not produce an additional decrease in both FOP and control cells. In agreement with our previous findings, *INHBA* expression was increased in FOP cells compared to controls. Following transdifferentiation, *INHBA* expression in FOP cells showed a significant decrease by day 14. Treatment with the inhibitor led to a further reduction in *INHBA* levels, with a statistically significant decrease compared to undifferentiated FOP cells at both timepoints (Table S1, Figure 4) [22].

**Figure 5:**
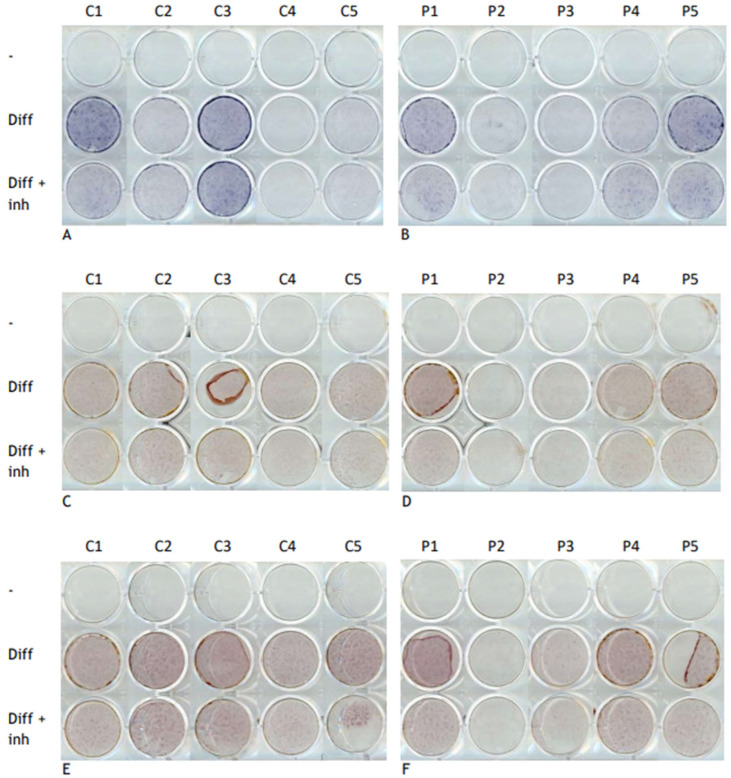
Effect of inhibitor on mineralization and ALP activity of FOP cells. ALP staining was conducted after 21 days (**A**,**B**) in cells cultured in control media (-), osteogenic media (diff), or in combination with the 223 nM inhibitor (diff + inh); AR staining was conducted after 21 (**C**,**D**) and 28 (**E**,**F**) days. Panels on the left show the five controls (C1–C5) and panels on the right show the five patients (P1–P5).

**Figure 6:**
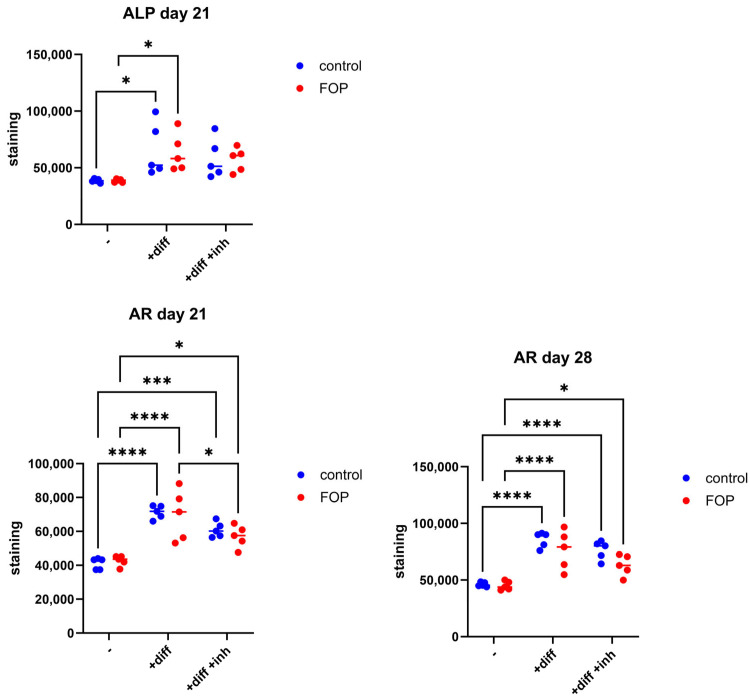
Quantification of ALP and AR staining in five FOP (P1–P5) and five control (C1–C5) cells cultured in control media (-), osteogenic media (diff), or in combination with the 223 nM inhibitor (+diff +inh). AR staining is significantly lower in FOP cells treated with the inhibitor compared to osteogenic media on day 21. Circles represent values for each cell line, lines represent medians, and significant differences are indicated with an asterisk (* *p* < 0.05, *** *p* < 0.001, **** *p* < 0.0001).

In order to study further the effect of the inhibitor on the mineralization process in FOP, ALP and AR stainings were employed after osteogenic transdifferentiation (Figure 5). The quantification of staining showed an increase in both FOP and control cells following treatment with osteogenic media compared to standard culture media; this was observed for ALP on day 21 as well as AR on days 21 and 28 (Figure 6). ALP activity was not significantly different between inhibitor-treated and untreated differentiated cells mirroring the lack of significant differences in *ALP* expression levels after inhibitor treatment (Figure 4). AR staining revealed a pattern of reduced average staining following treatment with 100 ng/mL inhibitor, with a significant reduction observed in FOP cells on day 21; however, staining levels remained elevated compared to the untreated condition both in FOP and control cells on days 21 and 28. Overall, no pronounced differences were observed between the FOP and control cells except for the decrease in AR staining levels on day 21 following inhibitor treatment.

## 4. Discussion

This study investigated the potential of the selective ALK2 inhibitor KTI-2338 in human FOP fibroblasts and transdifferentiated osteogenic cells, with the aim of suppressing hyperresponsive signaling that drives HO in FOP. Considering the established role of ALK2-related signaling in FOP, diffuse intrinsic pontine glioma and other pathologies, the persisting quest for ALK2 inhibitor advancement necessitates their validation in disease-relevant models [23]. Since the discovery of Dorsomorphin, explorations of chemical structures have strived to improve ALK2 inhibitor specificity [24]. Given the more demanding logistics of iPSC use, the newly developed KTI-2338 ALK2 inhibitor was tested in a model of primary human FOP fibroblasts that was previously shown to reflect the molecular consequences of the FOP *ACVR1* defect [22]. KTI-2338 successfully reduced osteogenic gene expression in both fibroblasts and transdifferentiated osteoblast-like cells, highlighting its potential as a therapeutic agent to inhibit the pathological processes driving HO formation in FOP.

Our findings show that the compound is able to inhibit the phosphorylation of SMAD1/5/9 and strongly reduces gene expression levels of *ID1* and *ID2* in FOP cells induced by Activin A. This indicates that the compound is able to prevent the stimulation of the BMP pathway in response to Activin A binding to the altered ACVR1 receptor in FOP cells. The compound did not show toxicity over the course of 21 days, corroborating that the reduction in pSMAD1/5/9, and of its transcriptional targets *ID1* and *ID2*, is likely specific to ACVR1 receptor inhibition and not due to cell toxicity. Furthermore, after the transdifferentiation of FOP fibroblasts into osteogenic cells, the inhibitor reduced the expression of *RUNX2* regulated by pSMAD1/5/9. There was also a pattern of lower AR staining levels in cells treated with the inhibitor with a significant decrease on day 21, specifically in FOP cells. These findings suggest that the compound successfully targets the overactive BMP pathway in FOP through the binding of the R206H variant ACVR1 receptor, supporting its potential application as an effective new therapeutic option to prevent HO formation in FOP.

Previous findings demonstrate the responsiveness of primary FOP fibroblasts to BMP4 and Activin A, which is exemplified by the upregulation of *ID1* and *ID2* SMAD1/5/9-responsive genes [22]. In line with this, treatment with Activin A upregulated the *ID1* and *ID2* expression of FOP fibroblasts. The expression of these genes has been found to be associated with osteogenesis; therefore, Activin A stimulation of *ID1* and *ID2* could represent the initiation of an osteogenic program in these cells. However, *ID1* expression decreased in both FOP and control fibroblasts following osteogenic transdifferentiation for 14 and 21 days. Both *ID1* and *ID2* are key targets of BMP-SMAD signaling; *ID1* is known to promote osteogenesis by enhancing *RUNX2* expression, whereas *ID2* regulates the early proliferation of progenitor cells into osteoblasts [25,26,27]. During the course of osteogenic transdifferentation, fibroblasts start to evolve osteoblast-like properties such as the ability to mineralize [28]. Thus, it can be hypothesized that the expression of very early osteogenic genes, such as *ID1*, decreases as the cells commit to the osteogenic lineage reflected at the later stages in our model. Notably, even though *RUNX2* and *ALP* are considered to be early markers of mesenchymal stem cell osteogenic differentiation, their upregulated expression was still detectable on days 14 and 21 [29,30]. Fibroblasts and mesenchymal stem cells share many properties; they are both adherent cell types with multipotent differentiation potential expressing CD73 and CD105 markers [31]. Despite the phenotypic fidelity of fibroblasts and their diverse use in osteogenic modeling, gene expression profile differences exist, which may account for variable expression in the induced osteoblast-related genes [32]. Additionally, variations in age and flare-up activity among donors could potentially impact gene expression levels [33,34]. Reported differences in Activin A expression in FOP cells can also contribute to variability in the observed outcomes and response to the KTI-2338 inhibitor [22]. FOP ACVR1 inhibition by the KTI-2338 inhibitor during osteogenic transdifferentiation maintained the low expression of *ID1*, which correlated with a significant decrease in *RUNX2*, suggesting that the compound can specifically interfere with the overactive ACVR1 receptor in FOP, in order to prevent the pathogenic osteogenic differentiation of progenitor cells.

Given the pivotal role of Activin A as the driver molecule of FOP pathology, its expression was interrogated in our model. Our earlier study showed that FOP fibroblasts produce Activin A, the expression of which is stimulated by TGFbeta superfamily members such as BMP4 and TGFβ1 [22]. In agreement with our former findings, *INHBA* gene expression reduced during osteogenic transdifferentiation in FOP cells, which may again reflect the alteration in cell properties during the gradual osteoblast maturation of the fibroblasts. TGFβ1 has been identified as a potent inducer of Activin A [22,35]. The high content of TGFβ1 in the platelet lysate, to which the fibroblasts are exposed during osteogenic transdifferentiation, indicates that TGFβ1 may fail to upregulate Activin A in more mature osteoblast-like FOP cells [21]. During this process, the KTI-2338 inhibitor did not influence *INHBA* levels.

Mineralization is a functional property of osteogenic cells, which is also reflected in our osteogenic transdifferentiation FOP model [21,28]. At the end of osteogenic induction, FOP cells showed significantly higher mineralization as assessed by AR staining; ALP activity was significantly increased in both FOP and control cells, which correlated with higher *ALP* gene expression levels. Interestingly, ACVR1 kinase inhibition by KTI-2338 significantly reduced mineralization in a FOP-specific manner, whereas ALP activity and expression were unaffected. Increased mineralization has also been documented in variable human cell FOP models [36,37,38]. Regarding ALP activity, connective tissue progenitor cells from exfoliated teeth of FOP patients and control individuals showed increased ALP activity after osteogenic induction, whereas negligible differences were observed between FOP and control periodontal ligament fibroblasts [39]. Although a decrease in mineralization has been reported in FOP cells following ACVR1 inhibition, this has been less investigated in relation to ALP activity (36). ALP activity was decreased in FOP iPSCs after an AAV-mediated CVR1R206H gene replacement approach [40]. Given the intrinsic differences between human FOP models, the biological significance of this outcome remains unclear in our model.

Currently, several selective small-molecule ALK2 inhibitors are being investigated in phase 2 trials as potential therapies for FOP, including the STOPFOP study of Saracatinib (NCT04307953), the FALKON study of IPN60130/Fidrisertib (NCT05039515), and the PROGRESS study of INCB000928/Zilurgisertib (NCT05090891). The FOP-repurposed Saracatinib was tested in vitro with an optimized iPSC model based on the differentiation of urinary cell-derived iPSCs into endothelial cells (iEC), which showed induced pSMAD1/5 expression in response to Activin A [41]. Although this might be an appropriate cell model to study drug potency, it entails a complex procedure with previous models showing reduced cell viability [38]. Fidrisertib and Zilurgisertib have not yet been reported to be studied in vitro in the context of FOP although the in vivo efficacy of Fidrisertib has been reported in a conditional knock-in ALK2R206H mouse model [42]. Another human primary cell model has been used to evaluate the potency of ALK2 macrocycle kinase inhibitors, using peripheral blood-derived endothelial colony-forming cells from FOP patients [36]. Considering the ubiquitous expression of ACVR1 receptors and ligands, the use of diverse models can help us validate the efficacy of emerging interventions and gain insight on potential side effects in order to facilitate optimal clinical transition. To this end, human cell models adequately recapitulating the disease pathology can be of value.

This study evaluated the novel compound KTI-2338 selectively targeting the ALK2 receptor in FOP. By assessing its effects in both FOP fibroblasts and their osteogenic progeny, we demonstrated a successful reduction in osteogenic signaling and mineralization. These findings suggest that the inhibitor may have therapeutic potential in preventing HO formation in FOP, adding to the therapeutic repertoire of ACVR1 signaling inhibitors in development. Additionally, this study highlights the value of our patient-derived primary cell model, effectively recapitulating pathological ACVR1 signaling in response to Activin A, as a platform to study new therapeutic interventions.

## Figures and Tables

**Table 1:** Fibroblast line donor characteristics. N/a—not applicable.

Cell Line	Diagnosis	Gender	Age at Biopsy	Flare-Up < 1 Week Prior to Biopsy	Medication at Biopsy
P1	FOP	M	23	No	No
P2	FOP	F	21	Yes	Celecoxib
P3	FOP	M	62	No	No
P4	FOP	M	14	Yes	Naproxen
P5	FOP	F	39	Yes	Tramadol
C1	Control	F	48	N/a	Unknown
C2	Control	M	44	N/a	Unknown
C3	Control	M	33	N/a	Unknown
C4	Control	Unknown	54	N/a	Unknown
C5	Control	M	29	N/a	Unknown

**Table 2:** Sequences of primers used for qPCR. F—forwards strand; R—reverse strand.

Gene	NM Number	Sequence	Strand	Tm	Product Length (bp)
*ALP*	NM_000478	AGGGACATTGACGTGATCAT CCTGGCTCGAAGAGACC	F R	56.62 56.21	242
*RUNX2*	NM_001024630.4	ATGCTTCATTCGCCTCAC ACTGCTTGCAGCCTTAAAT	F R	60.67 60.23	105
*ID1*	NM_002165	AATCCGAAGTTGGAACCCCC AACGCATGCCGCCTCG	F R	59.96 60.91	104
*ID2*	NM_002166	GTGGCTGAATAAGCGGTGTTC CTGGTATTCACGCTCCACCT	F R	59.87 59.46	371
*INHBA*	NM_002192	GTTTGCCGAGTCAGGAACAG TCACAGGCAATCCGAACGTC	F R	59.13 60.67	360
*YWHAZ*	NM_001135701.2	GATGAAGCCATTGCTGAACTTG CTATTTGTGGGACAGCATGGA	F R	58.49 58.00	229

## Data Availability

The data presented in this study are available on request from the corresponding author. Access to the data is restricted to protect participant privacy.

## Supplementary Materials

The following supporting information can be downloaded at https://www.mdpi.com/article/10.3390/pharmaceutics17121590/s1: Figure S1: Structures of LDN-193189 and KTI-2338; KTI-2338 In-vitro Potency/Selectivity; Table S1: Statistical analyses of gene expression after 14 and 21 days.
