# Supplementary material for: Compound KTI-2338 Inhibits ACVR1 Receptor Signaling in Fibrodysplasia Ossificans Progressiva
Source: Pharmaceutics. 2025 Dec 10;17(12):1590. doi: 10.3390/pharmaceutics17121590 (PMC12736380; doi:10.3390/pharmaceutics17121590)
Supplement: Supplementary file 1 [file pharmaceutics-17-01590-s001.zip › pharmaceutics-3997698-supplementary.pdf]

pharmaceutics

Compound KTI-2338 Inhibits ACVR1 Receptor Signaling in Fibrodysplasia Ossificans Progressiva

Supplementary material

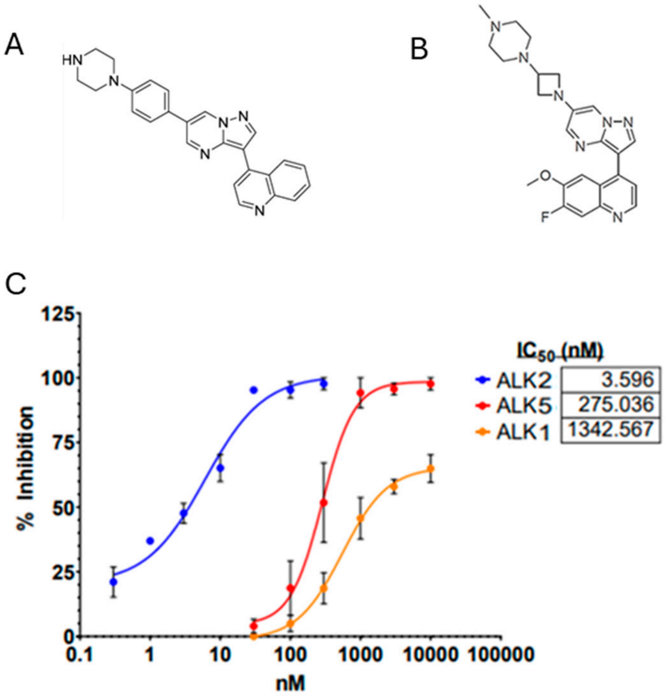

Figure S1. Structures of LDN-193189 (**A**) and KTI-2338 (**B**). KTI-2338 In-vitro Potency / Selectivity (**C**). A luciferase-based assay was used to determine IC50 values of KTI-2338 for ALK2, ALK5 and ALK1.

Table S1. A. *RUNX2* expression after 14 days in normal cells compared to FOP cells, calculated with Šídák's multiple comparisons test.

RUNX2	*Mean Diff,*	*95,00% CI of diff,*	*Summary*	*Adjusted P Value*
-	-0.0001772	-0.01374 to 0.01339	ns	>0.9999
+ diff	-0.01553	-0.02910 to -0.001963	*	0.0214
+ diff + inh	-0.002328	-0.01590 to 0.01124	ns	0.9619

Table S1. B. *INHBA* expression after 14 days in normal cells compared to FOP cells, calculated with Šídák's multiple comparisons test.

INHBA	*Mean Diff,*	*95,00% CI of diff,*	*Summary*	*Adjusted P Value*
**-**	-0.1295	-0.2252 to -0.03373	**	0.0059
+ diff	-0.03128	-0.1270 to 0.06445	ns	0.7947
+ diff + inh	0.002634	-0.09310 to 0.09836	ns	0.9998

Table S1. C. *ALP* expression after 14 days in normal cells compared to FOP cells, calculated with Šídák's multiple comparisons test.

ALP	*Mean Diff,*	*95,00% CI of diff,*	*Summary*	*Adjusted P Value*

-	-6.010e-005	-0.01707 to 0.01695	ns	>0.9999
+ diff	0.001224	-0.01578 to 0.01823	ns	0.9970
+ diff + inh	-0.002298	-0.01930 to 0.01471	ns	0.9807

Table S1. D. *ID1* expression after 14 days in normal cells compared to FOP cells, calculated with Šídák's multiple comparisons test.

ID1	***Mean Diff,***	***95,00% CI of diff,***	***Summary***	***Adjusted P Value***
-	0.02049	-0.01985 to 0.06083	ns	0.4971
+ diff	2.500e-005	-0.04031 to 0.04036	ns	>0.9999
+ diff + inh	-5.840e-005	-0.04039 to 0.04028	ns	>0.9999

Table S1. E. *RUNX2* expression after 21 days in normal cells compared to FOP cells, calculated with Šídák's multiple comparisons test.

RUNX2	***Mean Diff,***	***95,00% CI of diff,***	***Summary***	***Adjusted P Value***
-	-0.0002553	-0.007040 to 0.006529	ns	0.9996
+ diff	-0.008050	-0.01483 to -0.001266	*	0.0166
+ diff + inh	-0.001223	-0.008008 to 0.005562	ns	0.9563

Table S1. F. *INHBA* expression after 21 days in normal cells compared to FOP cells, calculated with Šídák's multiple comparisons test.

INHBA	***Mean Diff,***	***95,00% CI of diff,***	***Summary***	***Adjusted P Value***
-	-0.1007	-0.1815 to -0.01997	*	0.0115
+ diff	-0.03134	-0.1121 to 0.04943	ns	0.6983
+ diff + inh	0.009720	-0.07105 to 0.09049	ns	0.9862

Table S1. G. *ALP* expression after 21 days in normal cells compared to FOP cells, calculated with Šídák's multiple comparisons test.

ALP	***Mean Diff,***	***95,00% CI of diff,***	***Summary***	***Adjusted P Value***
-	-0.0002967	-0.03882 to 0.03823	ns	>0.9999
+ diff	0.003184	-0.03534 to 0.04171	ns	0.9954
+ diff + inh	-0.008664	-0.04719 to 0.02986	ns	0.9201

Table S1. H. *ID1* expression after 21 days in normal cells compared to FOP cells, calculated with Šídák's multiple comparisons test.

ID1	***Mean Diff,***	***95,00% CI of diff,***	***Summary***	***Adjusted P Value***
-	0.02152	-0.009305 to 0.05234	ns	0.2361
+ diff	-0.001797	-0.03262 to 0.02903	ns	0.9984
+ diff + inh	-0.0002122	-0.03103 to 0.03061	ns	>0.9999
